# Supplementary material for: Systemic Causes of In-Hospital Intravenous Medication Errors: A Systematic Review
Source: J Patient Saf. 2020 Feb 3;17(8):e1660–8. doi: 10.1097/PTS.0000000000000632 (PMC8612891; doi:10.1097/PTS.0000000000000632)
Supplement: SUPPLEMENTARY MATERIAL [file pts-17-e1660-s001.docx]

**Supplementary file 1. Description of the included studies (n=11): study designs, objectives, materials, main measures and key findings.**

| **Reference and country** | **Study design (evidence quality) and setting** | **Study objective** | **Materials and main measures** | **Key findings** |
| --- | --- | --- | --- | --- |
| Franklin et al. 2014 ^19^  United Kingdom | Retrospective analysis of medication error reports (Low), oncology hospital setting | To examine hazards related to vinca alkaloids, identify the defences breached and consider safety implications | ﻿Nine million patient safety incident reports were searched for medication error reports relating to administration of vinca alkaloids in patients also receiving intrathecal medication (n=38).  1) Severity of errors (degree of harm)  2) Process phase in which the error occurred  3) The principal defence(s) that had been breached for each incident | 1) None of the reports (n=38) resulted in actual harm.  2) The stage of medication process most commonly involved was ‘supply, transport and storage’ (n=15). Seven cases related to dispensing, six to documentation, and four to prescribing and administration.  3) Separation of intravenous vinca alkaloids and intrathecal medication in timing (n=16) and location (n=8); potential for confusion therefore remains. Problems involved in six cases did not align with the procedural defences in place. |
| Hicks and Becker 2006 ^24^  United States | Retrospective analysis of medication error reports (Low), hospital setting | To examine intravenous medication errors and why errors occur in the clinical setting | A machine count-determined convenient sample of intravenous medication errors reported to MEDMARX over a 5-year period (n=73 769).  1) Severity of errors (NCC MERP Index for Categorizing Medication Errors)  2) Error type  3) Causes of errors | 1) The amount of intravenous medication errors resulting in patient harm (3-5 %) was every year higher than all errors in administration routes.  2) The leading type of error was omission, and the leading cause involved clinician performance deficit.  3) Drug shortages (e.g. patient receives a sound-alike medicine because of the shortage), interconnectivity of tubings (e.g. switch in peripheral IV-line and epidural line, unlabelled tubing and syringes) and mistakes in calculations (e.g. 10-fold variance in paediatric medications) predispose patients to harmful errors. |
| Hicks et al. 2007 ^23^  United States | Retrospective analysis of medication error reports, (Low), neonatal intensive care unit (NICU) setting | To study medication errors associated with intralipid administration | Medication errors reported to MEDMARX over a 5-year period were searched to find errors associated with intralipid administration in NICU (n=257).  1) Severity of errors (NCC MERP Index for Categorizing Medication Errors)  2) Time of the error (day of week, time of error)  3) Error type  4) Causes of errors  5) Contributing factors to medication errors | 1) ﻿3.9% of errors were harmful.  2) ﻿ The largest number of errors occurred on Mondays﻿  (24.1%) and between 6 pm and midnight (28.8%).  3) ﻿Leading types: improper dose/quantity/amount (69.9%), wrong administration technique (11.3%), omission (8.6%).  4) ﻿The most common: improper pump use (27.1%), performance deficit (26.6%) and procedure protocol not followed (10.9%). Many errors resulted from misinterpretation of the modes (i.e. time, volume or rate) on infusion device or by not recognising decimal point on the device’s display panel. Several errors involved switching the rate of infusion with total parenteral nutrition and intralipids.  5) ﻿38.9% of error reports identified at least one contributing factor. The most common ones were none (42%), distractions (23.6%), and workload increase (11.3%). |
| Hicks et al. 2008 ^22^  United States | Retrospective analysis of medication error reports (Low), hospital setting | To study the magnitude, frequency, and nature of medication errors associated with patient-controlled analgesia (PCA) | Medication errors reported to MEDMARX over a 5-year period were searched to find errors associated with PCA (n=9 571, 1% of all error reports).  1) Severity of errors (NCC MERP Index for Categorizing Medication Errors)  2) Process phase in which the error occurred  3) Error type  4) Causes of errors  5) Contributing factors to medication errors | 1) 6.5% of errors were associated with harm.  2) Most errors occurred during drug administration, but errors in prescribing and dispensing stages were also identified.  3) 38% involved an improper dosage or quantity, 17.4% omission and 17.3% an unauthorised or wrong drug.  4) Human factors were the main cause of PCA errors. Equipment issues (19.5%) and similar drug names and product packaging (11.6%) were also implicated.  5) ﻿37.6% of error reports identified at least one contributing factor. The two leading ones were distraction (37.8%) and workload increase (19.7%). |
| Aguado-Lorenzo et al. 2013 ^18^  United Kingdom | Observational controlled study involving the analysis of infusion concentrations (Low), NICU and hospital pharmacy setting | To investigate accuracy of morphine infusion concentrations and to identify differences between preparation in NICU and pharmacy | ﻿Unused portions of morphine infusion solutions were collected over a 6-week period (n=214). They were prepared either in the pharmacy (n=115, 54%) or by nurses in the ward (n=99, 46%).  1) Concentration accuracy of prepared infusion solution according to the British Pharmacopoeia (±7.5%).  ﻿2) The frequency and magnitude of deviations found in infusions prepared in the pharmacy vs. NICU. | 1) ﻿19.2% of infusions prepared by nurses in the ward and 7.8% prepared in the pharmacy were outside the required limit. A deviation of more than 20% was found in ward-prepared infusions, although this was caused by volume discrepancies of less than 0.2 mL.  2) The frequency and magnitude of deviations in infusions prepared in pharmacy was lower than in those prepared by NICU. The latter showed a significantly higher number of out-of-specification samples (p=0.015); however, deviations occurred in both settings. Significant differences between pharmacy and NICU for volumes of <0.5 mL or <1 mL were not identified, probably due to small sample size. |
| Campino et al.  2016 ^25^  Spain | Observational controlled study involving the analysis of infusion concentrations (Low), NICU and hospital pharmacy setting | To assess the rate and causes of errors in intravenous medicine preparation at bedside in NICUs vs. the preparation error rate in hospital pharmacy | A total of 522 samples of prepared intravenous medications were collected prospectively: 444 from NICUs (study group) and 60 from hospital pharmacy (control).  1) Identification of calculation errors based on deviation from the intended concentration (±10%)  2) Identification of accuracy errors based on deviation from the intended concentration (±10%) | 1) Calculation errors were detected in 6/444 (1.35 %) samples prepared in NICUs. Three calculation errors came from NICUs that did not use standard concentrations. No calculation errors were detected in hospital pharmacy samples.  2) Accuracy errors were detected in 243/444 (54.7 %) samples prepared in NICUs and in 23/60 (38.3 %) samples prepared in hospital pharmacy. Weak points detected in the video analysis were the use of oversized syringes, failure to achieve good mixing and lack of volume control (vials or ampoules fully loaded) in NICUs and an insufficient mixing time in the hospital pharmacy. |
| Donaldson et al.  2011 ^21^  United Kingdom | Observational controlled study involving the analysis of infusion concentrations (Low), hospital ward setting | To determine the safest way of preparing homogenous electrolyte solutions for parenteral infusion | ﻿400 mmol/l infusions of KCl (n=16) and MgSO_4_ (n=16) diluted from concentrated stock solutions in 0.9% NaCl in 50ml syringes or 100ml PVC-bags. Mixed by inverting 10 times or agitating vigorously with a ‘vortex’ for 60 s. Infused immediately or left to stand for 24 h.  1) Concentrations of potassium and magnesium measured at regular intervals during infusions according to the US Pharmacopeia (±10%)  2) The overall homogeneity of the infusions quantified by the coefficient of variation (﻿the ratio of the standard deviation of each infusion to the mean value of that infusion) | 1) ﻿ Most of the infusions showed <10% variation from the expected concentration regardless of preparation technique. In general, the infusions delivered from syringes were more heterogeneous than those delivered from PVC bags. The concentrations of four magnesium infusions in syringes were >10% more concentrated than expected; one had a concentration 21.8% (95% CI 20.5%-23.1%) greater than expected for the first 5 min of the infusion.  2) The coefficients of variation of the infusions given immediately were greater than those that had been left to stand. |
| Keers et al. 2015 ^20^  United Kingdom | Interview study (qualitative, semi-structured) (Low), hospital setting | To investigate the underlying causes of intravenous medication administration errors (MAEs) | **﻿**Interviews were conducted with nurses (n=20) using the critical incident technique, where they were asked to discuss perceived causes of MAEs that they had been directly involved with (n=21). Transcribed interviews were analysed using the framework approach and emerging themes were categorised according to Reason’s model of accident causation.  1) Active failures (including systemic causes of errors)  2) Error and violation provoking conditions (contributing factors to medication errors) | 1) MAEs contained 23 individual active failures: slips and lapses (n=11), mistakes (n=8) and deliberate violations of policy (n=4). MAEs ﻿ shared causal elements such as equipment design (e.g. look-a-like medicines), distractions, lack of sufficient knowledge, unavailability of support resources and ﻿a decision not to challenge or question another member of the healthcare team.  2) Each active failure was associated with a range of error and violation provoking conditions. The working environment was implicated when nurses lacked healthcare team support and/or were exposed to a perceived increased workload during ward rounds, shift changes or emergencies. Nurses frequently reported that the quality of dose-checking activities was compromised. |
| Kim et al. 2014 ^27^  Republic of Korea | Interview study (focus-group) (Low), hospital setting | To identify the causes of and problems associated with high-risk intravenous injection errors | A focus-group interview was carried out with 13 healthcare professionals (7 nurses, 3 doctors, 3 pharmacists) under the theme of ‘the causes and problems of errors relevant to high-risk intravenous injections conducted by hospital nurses.’  1) ﻿Causes of the errors associated with high-risk intravenous injections  2) ﻿Problems associated with high-risk intravenous injections | 1) Three causal categories responsible for errors emerged: human-related, injection procedure-related, and environmental factors.  2) Specifically, lack of knowledge and awareness of high-risk medications, carelessness in controlling the speed and drip of fluids, injecting wrong fluid, unclear verbal prescriptions from doctors, inappropriate medication management, a shortage of medicators, and a lack of injection experience were identified as the most common problems responsible for nurses’ errors when administering high-risk intravenous injections. |
| Nerich et al. 2010 ^26^  France | Prospective analysis of medication orders (Low), University oncology hospital setting | To determine the incidence of prescribing medication errors (PME) and to analyse PME related to antineoplastic treatment | All medication orders (n=14 854) over one year were analysed prospectively by a resident or pharmacist to identify PMEs. Prescribers were contacted to check and to confirm prescriptions. In case of error, the prescription was corrected.  1) Potential clinical impact (according to the Hatoum scale)  2) Error risk factors (including system-based causes) | 1) The error incidence was 1.5%, with significant or very significant clinical impact in 62.9% of cases. Death-threatening events were avoided in 3.7% of cases.  2) The most common type of error was related to antineoplastic drug dosage (61.0%): inadequate adaptation (43.1%), not taking alarms into account (16.1%), incorrect weight (0.9%), incorrect unit (0.9%). More than 20% of errors were directly linked to the prescribing medication order (choice of treatment, double-prescribing, prescription forgotten or not validated). |
| White et al. 2014 ^28^  Canada | Direct observational study (Low), oncology hospital setting | To examine current process of ambulatory intravenous chemotherapy and to identify factors contributing to preventable adverse drug events | 1-week field observations were conducted at observation sites (n=6) to identify end-to-end processes in clinic, pharmacy, and treatment areas. An adaptation of Healthcare Failure Mode and Effects Analysis was used to identify potential sources of error.  1) Potential causes of medication errors | 1) Three types of previously unrecognised preparation errors were uncovered. The incorrect volume or type of diluent could be used in reconstitution because quality checks are not in routine use or may not be effective. The incorrect medication could be selected and drawn into a syringe, but the correct vial is then shown to pharmacist during their check, because multiple drugs are stored in close proximity in biological safety cabinet. The wrong patient’s label could be applied to a mixed bag because labels are not always physically affixed to the bag. |
