# Supplementary material for: Systemic Causes of In-Hospital Intravenous Medication Errors: A Systematic Review
Source: J Patient Saf. 2020 Feb 3;17(8):e1660–8. doi: 10.1097/PTS.0000000000000632 (PMC8612891; doi:10.1097/PTS.0000000000000632)
Supplement: SUPPLEMENTARY MATERIAL [file pts-17-e1660-s002.docx]

**SYSTEMIC CAUSES OF IN-HOSPITAL INTRAVENOUS MEDICATION ERRORS: A SYSTEMATIC REVIEW**

Sini Kuitunen (corresponding author), MSc (Pharm), ﻿HUS Pharmacy, Hospital Pharmacy of Helsinki University Hospital (HUS) and PhD student, Clinical Pharmacy Group, Faculty of Pharmacy, University of Helsinki, Finland.

Ilona Niittynen, MSc (Pharm), Clinical Pharmacy Group, Faculty of Pharmacy, University of Helsinki, Finland

Marja Airaksinen, PhD (Pharm), Clinical Pharmacy Group, Faculty of Pharmacy, University of Helsinki, Finland

Anna-Riia Holmström, PhD (Pharm), Hospital Pharmacy of Helsinki University Hospital (HUS) and Clinical Pharmacy Group, Faculty of Pharmacy, University of Helsinki, Finland

**Correspondence:** Sini Kuitunen, MSc (Pharm), ﻿HUS Pharmacy, Hospital Pharmacy of Helsinki University Hospital (HUS); PhD student, Clinical Pharmacy Group, Faculty of Pharmacy, University of Helsinki, Finland. (e-mail: sini.kuitunen@hus.fi, tel. +358407704394)

**Conflicts of Interest and Source of Funding:** Sini Kuitunen had a PhD research grant from the University Pharmacy (Yliopiston Apteekki), which is a pharmacy corporation owned by the University of Helsinki, Finland. The authors disclose no conflict of interests.

**ABSTRACT**

**Objectives:** Delivery of intravenous medications in hospitals is a complex process posing to systemic risks for errors. The objective of this study was to identify systemic causes of in-hospital intravenous medication errors.

**Methods:** A systematic review adhering to PRISMA guidelines was conducted. We searched MEDLINE (Ovid), Scopus, Cinahl and EMB reviews for articles published between January 2005 and June 2016. Peer-reviewed journal articles published in English were included. Two reviewers independently selected articles according to a predetermined PICO-tool. The quality of studies was assessed using the GRADE system and the evidence analysed using qualitative content analysis.

**Results:** 11 studies from six countries were included in the analysis. We identified systemic causes related to prescribing (n=6 studies), preparation (n=6), administration (n=6), dispensing and storage (n=5) and treatment monitoring (n=2). Administration, prescribing and preparation were the process phases most prone to systemic errors. Insufficient actions to secure safe use of high-alert medications, lack of knowledge of the drug, calculation tasks, failure in double-checking procedures and confusion between look-alike sound-alike medications were the leading causes of intravenous medication errors. The number of the included studies was limited, all of them being observational studies and graded as low quality.

**Conclusions:** Current intravenous medication systems remain vulnerable, which can result in patient harm. Our findings suggest further focus on medication safety activities related to administration, prescribing and preparation of intravenous medications. This study provides healthcare organisations with preliminary knowledge about systemic causes of intravenous medication errors, but more rigorous evidence is needed.

**Keywords**: patient safety; medication safety; intravenous medications; medication errors; systemic cause; risk management; systematic review

**INTRODUCTION**

Intravenously administered drugs are associated with the highest medication error frequencies and more serious consequences to the patient than any other administration route ^1–3^. The bioavailability of intravenously administered medication is high, therapeutic dose range is often narrow and effects are hard to undo. Many intravenously administered drugs are high-alert medications, bearing a heightened risk of causing significant patient harm if used in error ^4^. For example, in intensive care, the most serious medication errors are associated with intravenously administered high-alert medications, such as catecholamines, insulin, electrolytes, opioids and parenteral nutrition ^5,6^.

Intravenous medication administration is a multistep process involving specific administration devices, information systems and many healthcare professionals with different work tasks and skills. This complex delivery process poses to safety risks if appropriate systemic defenses are not in place ^7–10^. Identification of the systemic causes of medication errors (e.g. the possibility to make mistakes in infusion pump programming or confusion between similar drug names and packages) highlights the weaknesses of current intravenous medication practices. This enables the development of medication processes by implementation of effective systemic defences to prevent medication errors (e.g. smart infusion pumps with error reduction software or effective means to prevent confusion between similar drug names and packages).

However, the systemic causes of errors throughout the intravenous medication process have not been systematically reviewed. Previous systematic reviews have focused on types and incidence of intravenous medication errors ^8^ or the effectiveness of smart infusion pumps as a systemic defence ^11^. These studies present important knowledge of the frequency of errors and effectiveness of a systemic defence, but they do not focus on medication safety issues throughout the in-hospital intravenous medication process. The aim of our study was to explore recent evidence of systemic causes of in-hospital intravenous medication errors to inform medication safety improvement activities.

**METHODS**

**Study design**

A systematic review of recent evidence on systemic causes of in-hospital intravenous medication errors was carried out following the PRISMA guidelines for undertaking and presenting systematic reviews ^12^. The quality of included studies was assessed according to the GRADE system ^13^. The included articles were analysed using qualitative content analysis ^14,15^.

**Search strategy**

A systematic literature search was performed in June 2016 on MEDLINE (Ovid), Scopus, Cinahl and EBM reviews covering the period from January 2005 to June 2016. This period was chosen to focus on the most recent evidence published in peer-reviewed journals. An example of the search strategy is presented in Table 1.

We divided the search terms into two themes (‘intravenous medication therapy’ and ‘medication errors’), both of which needed to appear in the included articles. The theme ‘medication error’ was chosen according to our study objectives to explore preventable adverse drug events, which occur as a consequence of errors in the medication process caused by omissions or commissions ^3,16^. ﻿The search strategy was completed with other terms similar to ‘medication error’ (Table 1), as inconsistency in terminology and definitions related to medication errors is widely known ^17^. A combination of themes ‘adverse drug event’ and ‘intravenous’ was also considered. It was not included to the final search strategy, because the combination resulted to significantly wider amount of citations with the emphasis on drug safety and adverse drug reactions without the objective on medication safety and medication use process. We supplemented the search with a manual search of the reference lists of the included articles to identify all relevant publications.

**Inclusion and exclusion criteria**

We applied a predetermined PICO-tool (participants, interventions, comparison and outcomes) to select studies for inclusion ^12^. A study was included if participants were hospitalised patients or the study utilised a patient scenario in a simulated hospital environment, and patients received intravenous medication. We decided to exclude studies conducted in ambulatory settings, such as home infusion chemotherapy, as we wanted to focus on in-hospital intravenous medication process. We also excluded studies focusing on multiple administration routes, if the findings related to intravenous administration route could not be reliably identified and extracted from the results. Comparison was not required. Studies applying measures associated with systemic causes resulting in medication errors or assessment of a system defence to prevent medication errors were included. Studies exploring unpreventable adverse drug events or only incidence and types of medication errors were excluded. Only English language articles were included. Peer reviewed journal articles utilising all methods and study designs were included.

**Study selection**

After the removal of duplicates, the search produced 1417 potentially relevant publications (Figure 1). Two reviewers (SK, IN) independently selected studies based on the titles. In case of disagreement, the article was included in the next phase in which the reviewers (SK, IN) independently selected studies based on the abstracts. Disagreements were resolved through discussion and consensus with a third reviewer (A-RH). The reviewers (SK, IN) independently selected studies based on full-texts of the remaining publications. The articles fulfilling inclusion criteria by both reviewers were included (n=36). Disagreements were resolved through discussion and consensus with the third reviewer (A-RH), which led to the inclusion of nine more articles. A total of 45 publications met the inclusion criteria. Following this, reference lists of the included articles were searched manually for relevant articles (n=12), giving us a total of 57 included studies.

We identified two major themes among the selected articles: systemic causes of in-hospital intravenous medication errors and systemic defences to prevent errors (Figure 1). The articles focusing on systemic causes of intravenous medication errors (n=11) are reported in this publication. Articles focusing on systemic defences to prevent intravenous medication errors are discussed in another publication.

**Data extraction and analysis**

Data extraction and analysis were carried out by one of the authors (SK), and the results were carefully reviewed by the other authors (IN, A-RH, MA). Study characteristics, country and setting, objectives, study design, materials and methods, key findings and quality of evidence were extracted to a table (Supplementary File 1, <http://links.lww.com/JPS/A243>). We assessed the quality of evidence using the GRADE system, which has four levels of evidence: very low, low, moderate and high ^13^. Evidence from randomised controlled trials (RCT) was graded as high quality and evidence that included observational data was graded as low quality. Factors which decreased the quality of evidence (e.g. study limitations and inconsistency of results) or increased the quality of evidence (e.g. ﻿large magnitude of effect) were also taken in account. Measures used in the articles concerning systemic causes of in-hospital intravenous medication errors were extracted to Table 2.

We analysed the contents of the included articles using qualitative content analysis to identify systemic causes, examples of errors and suggested systemic defences for error prevention (Table 3)^10,14,15^. We used Leape's classic analysis of medication errors as a foundation of our taxonomy ^10^. Because of the fast development in medication safety research during the past decades and the most important medication safety issues arising from the studies included in our systematic review, we had to make some modifications to the categorizations (Table 3, Table 4). As we wanted to identify the most crucial systemic risk-factors causing errors in the intravenous medication process, we defined a systemic cause as a system failure or an iterative error-prone process step or task, which can be replaced with safer system modifications (e.g. calculation tasks related to preparation can be removed by using standard concentrations of prefilled syringes). The findings were extracted and classified according to the error type and medication process stage, in which the error happened or could have been prevented. The systemic causes affecting more than one process stage were identified and presented in Table 4.

**RESULTS**

**Characteristics of the included studies (n=11)**

This systematic review is based on 11 peer-reviewed original articles (Supplementary File 1, <http://links.lww.com/JPS/A243>). The studies were conducted in the United Kingdom (n=4) ^18–21^, United States (n=3) ^22–24^, Spain ^25^, France ^26^, Republic of Korea ^27^ and Canada ^28^. All studies were carried out in hospital setting. Three studies were conducted in neonatal intensive care units ^18,23,25^ and three in adult oncology ^19,26,28^.

All of the included studies applied an observational study design (Supplementary File 1, <http://links.lww.com/JPS/A243>). Four of the studies were retrospective analyses of medication error reports ^19,22–24^, three were observational studies involving analyses of infusion concentrations ^18,21,25^, two were interview studies ^20,27^, one was a prospective analysis of medication orders ^26^ and one was a direct observation study ^28^. The three studies investigating infusion concentrations in order to detect preparation errors ^18,21,25^ utilised a controlled study design. More than one error detection method was used in two studies, of which one combined a video analysis of preparation technique and revision preparation protocols with analysis of infusion concentrations ^25^, and the other used interviews to complement direct observation ^28^. Six studies used self-reporting methods, such as voluntary medication error reporting ^19,22–24^ and interviews ^20,27^. The study limitations were not reported and their influence was not assessed in three studies ^18,21,27^. None of the included studies applied RCT design, which is why they were graded as low quality ^13^.

The measures used to identify and describe systemic causes of medication errors in the studies varied, but some shared measures were identified (Table 2). Actual or potential systemic causes of errors (n=7) and the principal systemic defences that had been breached by each incident (n=1) were used in studies focusing on a larger scale of errors in multiple process stages. Concentration accuracy of prepared infusion solution (n=3) was used to identify preparation errors in studies comparing different ways of preparing intravenous medications in order to identify error risk factors. Three of the studies also focused on contributing factors to medication errors ^20,22,23^.

**Systemic causes of medication errors and potential systemic defences for error prevention**

The studies identified systemic causes of intravenous medication errors related to prescribing (n=6 studies), preparation (n=6), administration (n=6), dispensing and storage (n=5) and treatment monitoring (n=2) (Table 3). The process stage with the most systemic error causes identified was administration ^19,20,22–24,27^. The manual adjustment of infusion rates for each patient is an especially high-risk task, which can lead to wrong dose errors ^22,23,27^. An infusion pump programming error can occur as a consequence of confusion between hours and minutes (e.g. 20 min instead of 20 h) ^23^, weight and volume (e.g. order 5 mg/10 min, programmed 5 mL/10 min) ^22^, decimals (e.g. order 0.5 mL/h, programmed 5.0 mL/h) ^22,23^, volume and time (e.g. 24 mL instead of 24 min) ^23^, syringe sizes (e.g. 20-mL intended, 30-mL used and programmed) ^23^ or two medications’ infusion rates ^23^.

In all of the studies (n=11), potential systemic defences for intravenous medication error prevention were suggested (Table 3). Error prevention strategies were presented in discussion sections of the articles; thus, their effectiveness was not measured. Overall, activities related to process standardisation, replacement of error-prone tasks with technological solutions and staff education were suggested to decrease possibilities of errors and improve error detection ^18,19,28,20–27^.

Some systemic causes enabled medication errors in more than one process stage (Table 3, Table 4). Insufficient actions to secure safe use of high-alert medications ^19,20,27^ and lack of knowledge of the drug ^18,20–22,25,28^ were identified as the two causes which affected the most process stages, followed by calculation tasks ^22,24,25^ and confusion between look-alike sound-alike medications (LASA) ^20,22,24,26^. The studies also pointed out that absence of a systemic defence, or an existing defence breaking down, can enable errors. For example, failure to review orders after prescribing or to double-check during the preparation and administration stages can let errors actually reach the patient ^20,26,28^.

**DISCUSSION**

To the best of our knowledge, this is the first systematic review to summarise systemic causes of intravenous medication errors in hospitals. We found a limited number of studies, all of them being observational studies not providing the most rigorous evidence. Current intravenous medication systems remain vulnerable, which can result in patient harm. According to the included studies, administration, prescribing and preparation are the process phases most prone to systemic errors. We found insufficient actions to secure safe use of high-alert medications and lack of knowledge of the drug two leading error causes in multiple process stages, followed by calculation tasks, failure in double-checking procedures and confusion between LASA medications.

Considering the issues related to high alert medications, the Institute for Safe Medication Practices (ISMP) recommends standardising the ordering, storage, preparation, and administration of high-alert medications and improving access to information about these drugs ^4^. Furthermore, healthcare organisations should use multidisciplinary teams to review more carefully and standardise the use processes of high-alert medications through risk management strategies, such as failure-mode and effects analysis (FMEA) and root-cause analysis of reported errors ^22,23^.

Calculation tasks were identified as a cause of wrong dose errors in multiple medication process stages ^22,24,25^. Paediatric and neonatal populations are at the highest risk for life-threatening calculation errors because of weight-based dosing and inadequate commercial products ^24,29,30^. Standard concentration procedures are an important way to improve intravenous medication safety ^18,25,31,32^. Calculation tasks can also be eliminated or secured by successful implementation of other systemic defences, such as smart infusion pumps utilising error-reduction software, dose conversion charts and decision support systems ^22,29,33^. In addition, smart infusion pumps can reduce errors related to manual pump programming, which we identified as a particular high-risk task ^11,31^ ^22,23,27^.

Manual independent double-checks are widely used in error identification, but the frequent poor quality of these procedures can enable medication errors ^4,8,20,28,29^. Safety of procedures relying on accuracy and awareness of an individual is easily jeopardised. Likewise, procedures which lack sensitivity to all potential error types are problematic ^20,28^. Some manual double-checks could relatively simply be replaced with more reliable technological solutions (e.g., barcode scanning), or even eliminated by reducing error-prone process steps (e.g., reducing preparation errors by using pre-prepared syringes or sealed systems requiring minimal manipulation before use) ^8,18,21,25,34,35^.

In our study, absence of a standardised order review protocol was identified as a risk factor for inheritance of prescribing errors in later process stages ^20,26^. To support safe prescribing, an order review by a clinical pharmacist combined with clinical decision support systems would be an optimal strategy for error reduction ^4,26,29,33,36–38^. Also, confusion between LASA medications can be particularly significant when high-alert medications are involved ^22,27,39,40^. In order to decrease errors related to LASA medications, use of Tall Man lettering (e.g., morphine and HYDROmorphone), safe storage, auxiliary labels and barcode medication administration systems should be considered ^4,39,40^.

Our study was conducted in accordance with the PRISMA checklist ^12^. We included only peer-reviewed articles in the analysis and assessed the quality of selected studies using the GRADE system ^13^. The literature search was restricted to articles published in English, thus, studies published in other languages were excluded. Even though intravenous medications are widely used in hospitals and associated with frequent and particularly serious errors ^1–3^, the number of studies included in our systematic review was limited. Many excluded studies focused on incidence and types of intravenous medication errors, with no emphasis to examine why the errors happened. We also excluded some studies focusing on multiple administration routes, if the findings related to intravenous administration route could not be reliably identified and extracted from the results. We needed to make some modifications to the error categorizations presented in Leape's classic analysis of medication errors ^10^, as we wanted to identify the most crucial systemic causes of intravenous errors to inform medication process development in hospitals.

Probably due to our study objectives, none of the included studies applied an RCT design; the data could not be summarised statistically. Only two studies used more than one error detection method, which has been recommended to discover representative information concerning medication errors ^41^. Especially self-reporting methods have been associated with lack of representativeness and the issue of under-reporting. We also found a lot of variation between study objectives, designs and measures, which is an area of development. It is probable that administration seems the most complex and error-prone process stage because it was the most widely studied. Especially the evidence related to errors in treatment monitoring was limited. Furthermore, some important areas, such as microbiological contamination related to preparation were not identified as this was not measured in any of the studies, even though it has been recognised as an area of improvement ^42^.

In addition to systemic causes of intravenous medication errors, our initial target was to explore contributing factors. However, this was not possible, because contributing factors were explored in only three studies with variable research strategies ^20,22,23^. There is a need for further studies to explore systemic causes of intravenous medication errors in other settings than inpatient care, as intravenous administration, such as home infusion chemotherapy, is becoming more common in ambulatory settings.

**CONCLUSIONS**

According to our study, insufficient actions to secure safe use of high-alert medications, lack of knowledge of the drug, calculation tasks, failure in double checking procedures and confusion between LASA medications are the leading systemic causes of intravenous medication errors. Current intravenous medication systems remain vulnerable. Our findings suggest further focus on medication safety activities related to administration, prescribing and preparation of intravenous medications. Process standardisation and implementation of effective systemic defences are essential in order to improve medication safety. Our study provides healthcare organisations with preliminary knowledge about systemic causes of intravenous medication errors, but more rigorous evidence is needed.

**ACKNOWLEDGEMENTS**

Sini Kuitunen had a PhD research grant from the University Pharmacy (Yliopiston Apteekki), which is a pharmacy corporation owned by the University of Helsinki, Finland. The funder had no input into the design, methods, data collection, analysis, or preparation of this paper.

**REFERENCES**

1. Westbrook JI, Rob MI, Woods A, et al. Errors in the administration of intravenous medications in hospital and the role of correct procedures and nurse experience. *BMJ Qual Saf*. 2011;20(12):1027-1034.

2. Keers RN, Williams SD, Cooke J, et al. Causes of medication administration errors in hospitals: A systematic review of quantitative and qualitative evidence. *Drug Saf*. 2013;36(11):1045-1067.

3. Krähenbühl-Melcher A, Schlienger R, Lampert M, et al. Drug-related problems in hospitals: a review of the recent literature. *Drug Saf*. 2007;30(5):379-407.

4. Institute for Safe Medication Practices. ISMP list of High-Alert Medications in Acute Care Settings. http://www.ismp.org/tools/highalertmedications.pdf. Published 2018. Accessed November 30, 2018.

5. Valentin A, Capuzzo M, Guidet B, et al. Errors in administration of parenteral drugs in intensive care. *BMJ*. 2009;338.

6. Thomas AN, Macdonald JJ. A review of patient safety incidents reported as ‘severe’ or ‘death’ from critical care units in England and Wales between 2004 and 2014. *Anaesthesia*. 2016;71:1013-1023.

7. Taxis K, Barber N. Causes of intravenous medication errors: an ethnographic study. *Qual Saf Heal Care*. 2003;12(5):343-347. http://qualitysafety.bmj.com/lookup/doi/10.1136/qhc.12.5.343.

8. Mcdowell SE, Mt-isa S, Ashby D, et al. Where errors occur in the preparation and administration of intravenous medicines : a systematic review and Bayesian analysis. *Qual Saf Heal Care*. 2010;19(4):341-345.

9. Reason J. Human error: models and management. *Bmj*. 2000;320(March):768-770.

10. Leape LL, Bates DW, Cullen DJ, et al. Systems analysis of adverse drug events. ADE Prevention Study Group. *JAMA J Am Med Assoc*. 1995;274(1):35-43.

11. Ohashi K, Dalleur O, Dykes PC, et al. Benefits and Risks of Using Smart Pumps to Reduce Medication Error Rates: A Systematic Review. *Drug Saf*. 2014;37(12):1011-1020.

12. Moher D, Shamseer L, Clarke M, Ghersi D, Liberati A, Petticrew M, Shekelle P SL. Preferred Reporting Items for Systematic Review and Meta-Analysis Protocols (PRISMA-P) 2015 statement. *Syst Rev*. 2015;4(1):1-9.

13. Guyatt GH, Oxman AD, Kunz R, et al. GRADE: What is “Quality of evidence” and why is it important to clinicians? *BMJ*. 2008;336:995-998.

14. Elo S, Kyngäs H. The qualitative content analysis process. *J Adv Nurs*. 2008;62(1):107-115.

15. Hsieh H-F, Shannon SE. Three Approaches to Qualitative Content Analysis. *Qual Healthc Res*. 2005;15(9):1277-1288.

16. National Coordinating Council for Medication Error Reporting and Prevention. About Medication Errors. https://www.nccmerp.org/about-medication-errors. Accessed November 28, 2018.

17. Lisby M, Nielsen LP, Brock B, et al. How are medication errors defined? A systematic literature review of definitions and characteristics. *Int J Qual Heal Care*. 2010;22(6):507-518.

18. Aguado-Lorenzo V, Weeks K, Tunstell P, et al. Accuracy of the concentration of morphine infusions prepared for patients in a neonatal intensive care unit. *Arch Dis Child*. 2013;98(12):975-979.

19. Franklin BD, Panesar SS, Vincent C, et al. Identifying systems failures in the pathway to a catastrophic event: An analysis of national incident report data relating to vinca alkaloids. *BMJ Qual Saf*. 2014;23(9):765-772.

20. Keers RN, Williams SD, Cooke J, et al. Understanding the causes of intravenous medication administration errors in hospitals: A qualitative critical incident study. *BMJ Open*. 2015;5(3):1-10.

21. Donaldson TM, Mani V, Wheeler DW. Factors affecting the concentration of electrolyte infusions prepared from stock solutions. *Postgrad Med J*. 2011;87(1024):83-88.

22. Hicks RW, Sikirica V, Nelson W, et al. Medication errors involving patient-controlled analgesia. *Am J Heal Pharm*. 2008;65(5):429-440.

23. Hicks RW, Becker SC, Chuo J. A summary of NICU fat emulsion medication errors and nursing services: Data from MEDMARX. *Adv Neonatal Care*. 2007;7(6):299-308.

24. Hicks RW, Becker SC. An overview of intravenous-related medication administration errors as reported to MEDMARX®, a national medication error-reporting program. *J Infus Nurs*. 2006;29(1):20-27.

25. Campino A, Arranz C, Unceta M, et al. Medicine preparation errors in ten Spanish neonatal intensive care units. *Eur J Pediatr*. 2016;175(2):203-210.

26. Nerich V, Limat S, Demarchi M, et al. Computerized physician order entry of injectable antineoplastic drugs: An epidemiologic study of prescribing medication errors. *Int J Med Inform*. 2010;79(10):699-706. http://dx.doi.org/10.1016/j.ijmedinf.2010.07.003.

27. Kim M, Seomun G. Errors in high-risk intravenous injections administered by nurses: The causes according to healthcare professionals. *Heal Sci J*. 2014;8(2):249-261.

28. White R, Cassano-Piché A, Fields A, et al. Intravenous chemotherapy preparation errors: Patient safety risks identified in a pan-Canadian exploratory study. *J Oncol Pharm Pract*. 2014;20(1):40-46.

29. Ameer A, Dhillon S, Peters M, et al. Systematic literature review of hospital medication administration errors in children. *Integr Pharm Res Pract*. 2015:153. https://www.dovepress.com/systematic-literature-review-of-hospital-medication-administration-err-peer-reviewed-article-IPRP.

30. Ghaleb MA, Barber N, Franklin BD, et al. Systematic review of medication errors in pediatric patients. *Ann Pharmacother*. 2006;40(10):1766-1776.

31. Institute for Safe Medication Practices. PROCEEDINGS FROM THE ISMP SUMMIT ON THE USE OF SMART INFUSION PUMPS : GUIDELINES FOR SAFE IMPLEMENTATION AND USE. In: ; 2009. https://www.ismp.org/sites/default/files/attachments/2018-06/ISMP Smart Pump Guidelines 2009.pdf.

32. Larsen GY, Parker HP, Cash J, et al. Standard Drug Concentrations and Smart-Pump Technology Reduce Continuous-Medication-Infusion Errors in Pediatric Patients. *Pediatrics*. 2005;116(1):e21-e25. http://pediatrics.aappublications.org/cgi/doi/10.1542/peds.2004-2452.

33. Manias E, Kinney S, Cranswick N, et al. Interventions to Reduce Medication Errors in Pediatric Intensive Care. *Ann Pharmacother*. 2014;48(10):1313-1331.

34. Evley R, Russell J, Mathew D, et al. Confirming the drugs administered during anaesthesia: A feasibility study in the pilot National Health Service sites, UK. *Br J Anaesth*. 2010;105(3):289-296.

35. Hertig JB, Degnan DD, Scott CR, et al. A Comparison of Error Rates between Intravenous Push Methods: A Prospective, Multisite, Observational Study. *J Patient Saf*. 2018;14(1):60-65.

36. Manias E, Williams A, Liew D. Interventions to reduce medication errors in adult intensive care : a systematic review. *Br J Clin Pharmacol*. 2012;74(October 2011).

37. Bedouch P, Sylvoz N, Charpiat B, et al. Trends in pharmacists’ medication order review in French hospitals from 2006 to 2009: Analysis of pharmacists’ interventions from the Act-IP© website observatory. *J Clin Pharm Ther*. 2015;40(1):32-40.

38. Hermanspann T, Schoberer M, Robel-Tillig E, et al. Incidence and Severity of Prescribing Errors in Parenteral Nutrition for Pediatric Inpatients at a Neonatal and Pediatric Intensive Care Unit. *Front Pediatr*. 2017;5(June):1-9. http://journal.frontiersin.org/article/10.3389/fped.2017.00149/full.

39. Tyynismaa L, Honkala A, Airaksinen M, et al. Identifying High-alert Medications in a University Hospital by Applying Data From the Medication Error Reporting System. *J Patient Saf*. 2017;00(00):1-8.

40. Institute for Safe Medication Practices. ISMP ’ s List of Confused Drug Names. https://www.ismp.org/sites/default/files/attachments/2017-11/confuseddrugnames%2802.2015%29.pdf. Published 2011. Accessed November 30, 2018.

41. Härkänen M, Turunen H, Vehviläinen-Julkunen K. Differences Between Methods of Detecting Medication Errors: A Secondary Analysis of Medication Administration Errors Using Incident Reports, the Global Trigger Tool Method, and Observations. *J Patient Saf*. 2016;00(00):1-9.

42. Hedlund N, Beer I, Hoppe-Tichy T, et al. Systematic evidence review of rates and burden of harm of intravenous admixture drug preparation errors in healthcare settings. *BMJ Open*. 2017;7(12):e015912.

Figure 1. Flowchart of the study.

Table 1. Search strategy for the MEDLINE (Ovid).

Table 2. Measures used to identify and describe errors in the included studies (n=11).

Table 3. Systemic causes if intravenous medication errors and potential systemic defences for error prevention identified in the included studies (n=11).

Table 4. The most crucial systemic causes resulting in intravenous medication errors in more than one medication process stage.

Supplementary file 1. Description of the included studies (n=11): study designs, objectives, materials, main measures and key findings.
